# Assessing the clinical impact and resource use of a 30-minute chlamydia and gonorrhoea point-of-care test at three sexual health services

**DOI:** 10.1177/20499361211061645

**Published:** 2021-12-02

**Authors:** Susie Huntington, Georgie Weston, Elisabeth Adams

**Affiliations:** Aquarius Population Health Limited, Unit 29 Tileyard Studios, London N7 9AH, UK; Aquarius Population Health Limited, London, UK; Aquarius Population Health Limited, London, UK

**Keywords:** chlamydia infections, diagnosis, gonorrhoea, point-of-care testing, sexually transmitted diseases

## Abstract

**Objectives:**

To assess clinical metrics and resource use of a 30-minute point-of-care test (POCT) for *Chlamydia trachomatis* (CT) and *Neisseria gonorrhoeae* (NG) compared to laboratory-based testing.

**Methods:**

Three English sexual health services (SHSs) were recruited as part of a study. Existing processes for CT/NG testing and treatment were assessed, and adaptions to incorporate the CT/NG POCT were developed during semi-structured interviews. Staff time and consumables data were collected by clinic staff prior to and following introduction of the POCT.

**Results:**

SHSs selected patient groups for whom the CT/NG POCT would be used. Testing and treatment process data were collected for 225 patients (n = 118 POC; n = 107 standard). The percentage of patients receiving unnecessary CT treatment was 5% (5/95) and 13% (12/93) for POC and standard care respectively. The average CT/NG pathway cost varied and was on average £61.55 for POC and £50.88 for standard care. For the two SHSs where the POCT was used during a patient’s visit, for standard and POC respectively, the average time to CT treatment was 10.0 and 0.0 days and to NG treatment, 0.3 and 0.0 days.

**Conclusion:**

Use of a 30-minute POCT at three SHSs yielded clinical benefits by reducing time to treatment and unnecessary CT treatment.

## Introduction

Chlamydia, caused by *Chlamydia trachomatis* (CT) and gonorrhoea, caused by *Neisseria gonorrhoeae* (NG) are among the most common sexually transmitted infections (STIs) worldwide with an estimated 127.2 million incident cases of chlamydia and 86.9 million cases of gonorrhoea according to the most recent World Health Organization (WHO) estimates.^1^ In England, all patients attending a sexual health service (SHS) for an STI screen or with symptoms indicating an STI are offered testing for chlamydia and gonorrhoea as are some patients attending for other reasons, such as contraception.^2^ In 2018, almost 2 million CT/NG diagnostic tests were performed in SHSs in England.^3^ There is typically some delay between sample collection and results notification when samples are sent offsite to a diagnostic laboratory. The turnaround time varies between SHSs and in some cases exceeds eight working days, the standard set by the British Association for Sexual Health and HIV (BASHH).^4–6^ Some patients are treated at their first attendance, particularly for CT. This is based on risk, where there is clinical indication, if the patient is a partner of a positive contact or is unable or unlikely to return to clinic for treatment. This reduces any potential onward transmission, minimises the need for return appointments, and reduces the risk of loss to follow-up (LFTU) but inevitably results in some patients receiving unnecessary or incorrect treatment.

There are a small number of rapid tests for CT, NG, or CT and NG available with one semi-rapid (90-minute) test with equivalent performance to laboratory nucleic acid amplification tests (NAATs).^7–9^ POCTs provide an opportunity for patients attending SHSs to be tested and treated at their first visit thereby reducing the time to results,^10–12^ time to treatment,^12–14^ and the proportion of patients receiving unnecessary syndromic/presumptive treatment.^10,15^ POCTs typically cost more than the equivalent laboratory tests but can result in overall cost savings to the health system by reducing return appointments, unnecessary treatments, staff time spent on results notification and follow-up, onward transmission, and disease sequalae.^11,16^ Many healthcare settings already use POC testing, for example for HIV testing, and these types of tests are typically viewed favourably by patients.^17,18^

There are no published data on how many UK SHSs are currently using CT/NG POCTs, but use is thought to be low. To maximise the benefit to patients and the SHS, adoption of these technologies requires an assessment of existing practice and a redesign of clinical pathways.^11,12^ Few examples exist of how SHSs have successfully adopted CT/NG POCTs or the impact on clinical outcomes and resource use.^12,16^

In 2019, the first 30-minute dual CT/NG NAAT (binx health *io*® CT/NG assay) was licenced for use in Europe and the United States.^19^ This assessment sought to measure the impact of replacing CT/NG laboratory testing with a 30-minute CT/NG POCT at three self-selected SHSs in England considering adopting POC testing. The work presented summarises the steps involved in CT/NG testing and treatment in both standard and POC pathways for specific patient groups and compares the clinical outcomes and resource use for each pathway.

## Methods

### Outcome measures

The steps involved in testing and treating CT/NG for standard and POC pathways were summarised as flow diagrams for each patient group at each SHS.

Several clinical outcomes were assessed:

1. Average number of days to CT/NG result – from sample collection to patient notification.
2. Average number of days to treatment – from first visit, in CT/NG positive patients.
3. Percentage of patients receiving unnecessary CT treatment – that is, treatment given at first attendance to CT-negative patients.
4. Percentage of patients receiving unnecessary treatment for NG – (see Outcome 3).
5. Percentage of patients lost to follow-up (LTFU) – that is, patient is CT/NG positive (laboratory or POCT) but does not receive treatment for the infection.
6. The resources used (staff time and consumables) and associated cost per patient for each CT/NG pathway.

### Pathway definition and perspective

For this evaluation, the pathway included all steps involved in testing and treating CT/NG from the patient’s first attendance at clinic (for this episode) to the patient either receiving a negative test result or treatment for the infection – which results in no further attendance. Only costs incurred by the SHS were considered; patients’ time and out of pocket expenses were not assessed.

### Clinic recruitment

As part of a wider collaborative research project and service evaluation with the Applied Diagnostic Research and Evaluation Unit at St George’s University of London, and binx health, SHSs were recruited to the project via the BASHH newsletter, the BASHH conference and direct invites to existing contacts. SHSs keen to participate were asked to host a workshop attended by their clinic and laboratory staff and members of the collaborative research project. These were used to explain the project, demonstrate the POCT and brainstorm how it could best be utilised within the service. Of the seven SHSs which hosted a workshop, three went on to participate.

### CT/NG POCT

The binx health *io*® CT/NG assay is a CE-marked, FDA cleared NAAT cartridge with 99% specificity (95% confidence interval (CI) 98.6–99.8%) and 96% sensitivity (CI 89.4–98.3%) for CT and 100% specificity (CI 99.6–100.0%) and 100% sensitivity (CI 93.0–100.0%) for NG (data for females).^20^ The binx health *io* instrument and cartridges are designed for use in clinic, using clinician or patient collected samples (swabs/urine). Sample material is mixed with buffer solution, added to the cartridge which is inserted into the desktop sized platform. It takes approximately 30-minutes to process the result which is displayed on the screen in a qualitative format. The platform can be linked to the clinics’ electronic patient record system. Following use, the cartridge is automatically sealed and is then disposed. Each *io*^®^ instrument runs one assay at a time, but multiple instruments can be used simultaneously. (For further details see https://mybinxhealth.com/poc-more-info/).

### Mapping CT/NG testing and treatment pathways

Adapting methods previously used to map CT/NG pathways in SHSs,^16^ audio-recorded semi-structured interviews with service leads and/or clinical staff were held at each clinic in June–December 2018. These were used to create an overview of steps and resource use for CT/NG testing and treatment. After discussing how the pathway could be adapted, the mapping process was repeated for the hypothetical POC pathway. (See online Supplementary Material Tables S1–S3 for further details of the approaches used for data collection and analysis).

Following the interview, summary diagrams were created in Microsoft Excel (Office 365, Microsoft, Redmond, USA) for each pathway – listing the resource use (staff time, diagnostics, consumables, and medication) for each step. These were reviewed by the main contact at each clinic and were later simplified (for this paper). (Unit costs are presented in Tables S4-S6).

### Data collection by SHSs to assess clinical outcomes and resource use

Following pathway mapping, data collection (paper) forms were developed for use by clinic staff to record data on resource use for each pathway. Separate forms were developed for collecting data at first/return attendance (Form A and Form B), additional tasks performed during an attendance (Form C), or following the attendance (Form D), at 30-days follow-up (Form E) and for clinic staff paygrade (Form F). Data collected on each form are listed in Table 1.

**Table 1.** Summary of data collection forms used by clinic staff to collect clinical and resource use data for comparing standard and POC CT/NG testing and treatment pathways.

Form	Purpose of the form	Data collected
A	First attendance – main (1 form per patient)	● Staff initials ● Patient’s unique project ID ● Patient group (e.g. contact, symptomatic) ● Sexual risk group (MSM, heterosexual men, and women) ● Reason for attendance (e.g. LARC and contact) ● Staff initials and role ● Time spent on each task performed ● Medication ● Pathology ● Diagnosis and method of diagnosis ● Consumables relating to tasks performed
B	Return attendance – main (0-1 form per patient)	● Same as Form A ● Plus ● Previous visits ● Medication at previous visit
C	Additional tasks during attendance (first or return) (0-1 form per patient)	● Same as Form D ● Plus ● Medication
D	Additional tasks following attendance (0-1 form per patient)	● Staff initials and role ● Patient’s unique project ID ● Time spent on each task performed ● Consumables relating to tasks performed
E	30-day follow-up (1 form per patient)	● Patient’s unique project ID ● Number and date of visit/s ● Reason for return visit/s ● If they should have returned but did not ● Diagnosis including CT/NG lab/POCT result ● Time to CT/NG test result
F	Staff pay grade (1 form per member of staff who completed Forms A to D)	● Staff initials ● Job title and grade^21^ ● Tasks performed (relating to CT/NG)

CT, chlamydia; LARC, long-acting reversible contraception; MSM, men who have sex with men; NG, gonorrhoea; POCT, point-of-care test.

Forms were completed between April and October 2019. Taking into account practical considerations and the needs of the service, each SHS decided on either the total number of forms they would complete or a specific period during which they would complete forms for all patients in their selected patient group/s. The patient groups were selected by the SHSs based on pragmatic reasons such as patient numbers and practical considerations and the perceived acceptability and benefit to those patients.

### Patient and public involvement

There was patient/public representation on the advisory committee for the wider collaborative project. These representatives provided input on the design of the pathway mapping and data collection exercise.

No unique patient identifying information or demographic data such as age or ethnicity were collected. Steps were taken to ensure clinic IDs were removed from forms before forms were sent to the research team (see Table S2). Information on gender/sexual risk (i.e. heterosexual or MSM (Men who have Sex with Men)) were collected – as this would impact pathway costs since different sample types or number of samples are collected for men/women and according to exposure risk.

### Data analysis

Data from the forms were entered into a Microsoft Access database and reviewed by a second individual to check for accuracy. Clinical outcomes and resource use data were then analysed in Excel. Data for patients were excluded if the patient had not been tested for CT/NG, where resource use data were missing or where no equivalent forms were completed in the standard/POC pathway for that patient group.

The average cost per patient for each pathway and each patient group was calculated using a micro-costing approach. The minutes spent on each activity by each staff grade were used to calculate the average staff cost for each pathway plus consumables used for sample collection, diagnosis, treatment, treatment notification, health promotion and medication for each attendance, plus any follow-up or return appointment.

Due to the small sample size, statistical analyses were not performed, and data for columns containing <5 individuals are not presented.

## Results

### Clinic recruitment

Initially, pathways were mapped at five SHSs where preliminary workshops had been hosted. Three SHSs then went on to participate in the project and collect resource data within their clinic/s – the results of which are presented here. The three participating SHSs, representing five clinical sites, differed in the characteristics of the local population, in the way they provide CT/NG testing and treatment, and in the patient groups they selected for CT/NG POC testing. The Supplementary Material contains detailed results for SHS 1 (Tables S7–S12), SHS 2 (Tables S13–S18), and SHS 3 (Tables S19–S24).

### Patient groups

The patient groups selected at each SHS are listed in Table 2. Further details about the clinics and patient groups are presented in Tables S7, S13, and S18.

**Table 2.** Patient groups and summary data for clinics where pathway and resource data were collected for CT/NG standard and POC pathways.

Sexual health service	SHS 1		SHS 2		SHS 3	
Patient groups selected	All attendees at drop-in clinics for <25 s and select groups from drop-in clinic for all ages.^a^		Symptomatic Contacts (asymptomatic) LARC^b^		Symptomatic Contacts (asymptomatic) Asymptomatic (hard to access services)	
Patients with pathway data collected^c^	Standard	POC	Standard	POC	Standard	POC
	38	46	19	24	50	48
Patient characteristics (standard and POC data combined)						
Total number	84		43		98	
Women (%)	34	(40%)	22	(51%)	55	(56%)
MSM (%)	5	(6%)	4	(9%)	6	(6%)
MSW (%)	30	(36%)	14	(33%)	28	(29%)
Other group or not reported (%)	15	(18%)	3	(7%)	9	(9%)
CT/NG prevalence (standard and POC data combined)d						
CT^e^ (%)	24	(29%)	10	(23%)	4	(4%)
NG^e^ (%)	5	(6%)	4	(9%)	0	(0%)
CT/NG co-infection (%)	2	(2%)	0	(0%)	0	(0%)

CT, chlamydia; LARC, long-acting reversible contraception; MSM, men who have sex with men; MSW, men who have sex with women; NG, gonorrhoea; POC, point-of-care; SHS, sexual health services.

a Includes: symptomatic, asymptomatic, contacts (symptomatic and asymptomatic), and contraception.

b LARC here refers to the intrauterine device and the intrauterine system.

c This does not include excluded forms: n = 53 at SHS 1 (Table S8); n = 2 at SHS 2 (Table S14) and n = 2 at SHS 3 (Table S20). Patient numbers and risk groups for selected patient groups where clinical outcome and resource use data were collected are presented in Tables S9, S15, and S21.

d This table includes only infections diagnosed using molecular testing (lab or POCT). CT and NG prevalence data for standard and POC pathways are presented for each SHS in Tables S10, S16, and S22.

e Includes those with CT/NG co-infection.

### Pathway changes to incorporate CT/NG POC testing

The pathways were summarised as diagrams and are presented in online Supplementary Material Figures S1–S6. A typical pathway change is shown in Figure S3. The return appointment for treatment is not required in the POC pathway since anyone with a positive result is treated at their first attendance. Patients still have laboratory tests for other infections (e.g. HIV and syphilis) and therefore receive results *via* text message for those tests. In some cases, samples were self-collected in the POC pathway instead of clinician collected in the standard pathway (Figure S1, S4, and S5).

In standard care, SHS 3 sent samples away to be tested at a central laboratory. Instead of using the rapid test during the patient’s attendance, they chose to wait and run the test on samples in the clinic at a later time when either a healthcare assistant or nurse had availability (Figure S6).

### Clinical outcomes

Clinical outcome data are presented in Table 3. For every SHS and for every patient group, the average time to CT/NG result notification was demonstrably shorter in POC than for standard pathways. The time to results notification was on average 7.9 days shorter for POC pathway than for standard care when data for all three SHSs were combined, and 6.6 days shorter when data for SHS 1 and 2 were combined (i.e. SHSs that used the test to give results during the attendance). For SHS 1 and 2, the delay between first attendance and treatment for patients with chlamydia was on average 10.0 days for standard care and 0.0 days for POC. For patients with gonorrhoea, the delay was 0.3 for standard care and 0.0 days for POC.

**Table 3.** Clinical outcomes for patients with data collected to compare the use of standard CT/NG pathways with POC pathways.

Pathway		SHS 1		SHS 2		SHS 3 ^a^		SHS 1 + 2 combined^a^		All SHSs combined	
		Standard	POC	Standard	POC	Standard	POC	Standard	POC	Standard	POC
Total	(A)	38	46	19	24	50	48	57	70	107	118
CT positive	(B)	11	13	3	7	1	3	13	20	15	23
Correctly treated at first appointment	(n/B)	3 (27%)	13 (100%)	3 (100%)	7 (100%)	1 (100%)	2 (67%)	6 (46%)	20 (100%)	7 (47%)	22 (96%)
Treated at return appointment	(n/B)	8 (73%)	0 (0%)	0 (0%)	0 (0%)	0 (0%)	1 (33%)	8 (62%)	0 (0%)	8 (53%)	1 (4%)
Average wait (days) for treatment^b^		11.2	0.0	0.0	0.0	0.0	0.0^c^	10.0	0.0	5.3	0.0^c^
CT negative	(C)	27	33	16	17	49	45	44	50	92	95
Unnecessary CT treatment^d^	(n/C)	2 (7%)	2 (6%)	6 (38%)	2 (12%)	4 (8%)	1 (2%)	8 (18%)	4 (8%)	12 (13%)	5 (5%)
NG positive	(D)	2	3	2	2	0	0	4	5	4	5
Correctly treated at first appointment	(n/D)	1 (50%)	3 (100%)	2 (100%)	2 (100%)	–	–	3 (75%)	5 (100%)	–	–
Treated at return appointment	(n/D)	1 (50%)	0 (0%)	0 (100%)	0 (100%)	–	–	1 (25%)	0 (0%)	–	–
Average wait (days) for treatment^b^		0.5	0.0	0.0	0.0	–	–	0.3	0.0	–	–
NG negative	(E)	36	43	17	22	50	48	53	65	103	113
Unnecessary NG treatment^d^	(n/E)	0 (0%)	0 (0%)	0 (0%)	0 (0%)	0 (0%)	0 (0%)	0 (%)	0 (%)	0 (0%)	0 (0%)
Average (mean) time to result (days)		7.9	0.0	4.2	0.0	15.5	6.5	6.6	0.0	10.3	2.4
Return appointment^e^	(n/A)	8 (21%)	5 (11%)	2 (11%)	4 (17%)	5 (10%)	9 (19%)	10 (18%)	9 (13%)	15 (14%)	18 (15%)
Lost to follow-up^f^	(n/A)	0 (0%)	0 (0%)	0 (0%)	0 (0%)	0 (0%)	0 (0%)	0 (0%)	0 (0%)	0 (0%)	0 (0%)

CT, chlamydia; NG, gonorrhoea; POC, point-of-care; SHS, sexual health services.

Cells are left blank where not applicable. Data for each patient group for standard and POC pathways are presented for each SHS in online Supplementary Material Tables S11, S17, and S23.

a The SHS 3 POC pathway is not a genuinely POC pathway, since samples were not tested during the patient’s first attendance; therefore, combined data are presented for SHS 1 and SHS 2. In POC at SHS 3, 9/48 of the patients received their result on their same day as they tested.

b Average wait (days) for treatment is average wait between first attendance and treatment being received for all patients diagnosed with chlamydia or gonorrhoea.

c For one patient at SHS 3, data were missing on the date the patient returned for CT treatment after receiving their CT/NG test result on the same day they attended.

d Unnecessary treatment is treatment given at the first appt for CT/NG with a subsequent negative test result. This does not include patients treated for nongonococcal urethritis (NGU).

e Return appointment for any reason.

f Lost to follow-up refers to patients diagnosed with chlamydia or gonorrhoea who are not reported as having received treatment for the infection within the 30-day follow-up period.

In current care, patients not treated at their first visit, who were then diagnosed with chlamydia, waited on average 9-days for their result and 14-days for treatment from first attendance.

For all clinics combined, the percentage of patients who received unnecessary CT treatment was 13% (12/92) in standard and 5% (5/95) in POC pathways (where the denominator is CT-negative patients). The percentage of patients who received correct treatment at their first attendance was 47% (7/15) in standard care and 96% (22/23) in POC (where the denominator is CT positive patients).

No patients received unnecessary treatment for NG in either standard or POC pathways, and there was no evidence that any patients were lost to follow-up.

### Resource use

When average pathway costs were combined, weighted according to the number of patients in that patient group among those in the evaluation, the overall average cost was £61.55 for POC and £50.88 for standard care – POC costing an additional £10.67 overall, £7.76 for SHS 1, £11.61 for SHS 2, and £9.90 for SHS 3 (Table 4).

**Table 4.** Average pathway costs for standard and POC CT/NG testing and treatment pathways – calculated using resource use data collected in clinic for specific patient groups.

Sexual health service		SHS 1^a^		SHS 2		SHS 3		All SHSs	
Pathway		Standard	POC	Standard	POC^b^	Standard	POC^b^	Standard	POC
Staff time	First visit main staff	£23.73	£31.08	£36.73	£37.78	£15.30	£14.22	£22.10	£25.58
	First visit additional staff	£1.69	£2.46	£5.61	£9.10	£4.12	£4.65	£3.52	£4.70
	Second visit main staff	£6.91	£2.57	£1.14	£1.63	£1.42	£2.67	£3.32	£2.42
	Second visit additional staff	£0.00	£0.00	£0.00	£0.00	£0.00	£0.00	£0.00	£0.00
	Follow-up^c^	£7.72	£0.00	£2.68	£2.38	£2.84	£2.18	£4.54	£1.37
	Total staff time cost	£40.04	£36.10	£46.16	£50.89	£23.68	£23.72	£33.48	£34.07
Consumables	First visit	£1.61	£1.94	£2.63	£3.05	£2.45	£2.40	£2.18	£2.35
	Second visit	£0.07	£0.08	£0.11	£0.13	£0.10	£0.19	£0.09	£0.13
	Follow-up^c^	£0.11	£0.00	£0.28	£0.27	£0.24	£0.28	£0.20	£0.17
Medication	First visit	£1.16	£3.10	£5.08	£0.94	£1.03	£0.22	£1.80	£1.49
	Second visit	£1.34	£0.37	£0.00	£0.00	£0.17	£0.32	£0.56	£0.27
Diagnostics	First visit	£12.51	£23.00	£12.66	£23.24	£12.58	£23.03	£12.57	£23.06
Total average pathway cost		£56.84	£64.60	£66.91	£78.52	£40.25	£50.15	£50.88	£61.55

CT, chlamydia; NG, gonorrhoea; POC, point-of-care; SHS, sexual health services.

Data for each patient group for standard and POC pathways are presented for each SHS in online Supplementary Material Tables S12, S18, and S24.

a There are likely to be some resource savings in POC at SHS 1 which were not captured. Clinics A and B refer patients to a central clinic when the patient requires microscopy or where NG or *Trichomonas vaginalis* (TV) is suspected. For practical reasons, referral rates data were not collected. This is likely to have underestimated the benefit of the POC test since some patients may have avoided referral.

b No data on resource use at return appointments were collected for the POC pathway for some patient groups who had a second attendance (reported on their 30-day follow-up form). To avoid under-estimating the cost of the POC pathway, the average cost per person per return visit (in standard pathway) was used to calculate the average cost of these return visits for the POC pathway and included in the total average cost of the pathway.

c Follow-up refers to the tasks performed following the attendance such as processing and communicating test results.

For SHS 1, staff time increased for some groups and reduced for others (Table S12) – on average, for the groups combined, staff time cost £40.04 for standard care and £36.10 for POC. The total cost was higher for POC than standard care for all patient groups except for emergency LARC (long-acting reversible contraception) (£16.99 saving).

For SHS 2, overall, POC cost £78.52 and standard care £66.91. The cost of POC was lower than standard care for two patient groups – emergency LARC (£7.99 saving) and symptomatic patients (£2.83 saving).

For SHS 3, where CT/NG pathways were not altered (Figure S6), standard pathway cost £40.25 and POC £50.15 per patient. This difference came from the increase in diagnostics cost (£12.58 for standard and £23.03 for POC) as the average staff time costs increased by only £0.04 (£23.68 for standard and £23.72 for POC) (Table S24).

## Discussion

Standard and POC CT/NG testing and treatment pathways were mapped in three SHSs serving distinct populations, and clinical and resource use data were collected in clinic. SHSs selected diverse patient groups for POC testing including groups more likely to have chlamydia and/or gonorrhoea, such as contacts and symptomatic patients, as well as lower-risk groups such as women receiving LARC.

This is the first study to use data collected in clinic to assess clinical outcomes and resource use at multiple SHSs comparing laboratory testing with a 30-minute NAAT for CT/NG with laboratory equivalent clinical performance. Previous studies report pathway changes in SHSs based on estimates, models, or used data from a single SHS.^12,16,22–25^ Data were collected for each step of the pathway, including follow-up, and even when multiple staff were working simultaneously, providing a more complete picture of resource use. However, the burden of data collection was on busy clinic staff whose priority is clinical work. Therefore, data were collected for a relatively small number of patients and as such, statistical and sensitivity analyses were not appropriate. It also meant that there was inconsistency in the data quality resulting in some exclusions.

The results of this analysis provide further evidence that CT/NG POC testing improves patient management, reducing the time to results and to treatment, and reducing unnecessary CT treatment – as have been reported in previous studies.^10–12^ At the two SHSs where the POCT was used to test and treat patients during their attendance, the average wait for results fell from 6.6 to 0.0 days. At SHS 3, where samples were not tested during the patients attendance, the time to results decreased from 15.5 to 6.5 days, below the 8-day threshold set by BASHH.^6^ There may have been some selection bias in which SHSs chose to participate in the overall study, for example, favouring SHSs frustrated by long turnaround times for results. This is likely to be the case for SHS 3; however, it is difficult to assess since results turnaround times are not routinely published. Expediting the time to results and treatment will likely result in fewer onward transmissions impacting prevalence within the population in the longer term.^22^ Avoiding inappropriate treatment improves antibiotic stewardship and is important in light of multi-antibiotic resistant strains of NG in the United Kingdom and elsewhere which pose a serious public health threat.^26^ There is currently no cost threshold used to assess the cost-effectiveness of avoiding use of antibiotics.

The number of patients returning to the clinic for treatment or for other reasons was very low in standard pathways, in part because the prevalence of CT/NG was low and because syndromic/presumptive treatment for chlamydia was often given (some of which was unnecessary). This meant there was only a small difference in the percentage of patients returning in standard and POC pathways (18% *vs* 13%, respectively for SHSs 1 + 2) and there was no or minimal cost savings in the staff time spent on return appointments. Overall, the average cost of the POC pathways was roughly £10 more than the cost of the standard pathways – equivalent to the estimated difference between the laboratory and POC test. However, for some patient groups, the average pathway cost reduced when the POCT was used due to savings in staff time. Although these were small sample sizes, this demonstrates that the difference in cost between standard and POC pathways differs between patient groups.

This assessment may underplay the benefit of the CT/NG POCT because there were likely to be other benefits which were not assessed including patient utility, reduced onward transmission and sequalae and reduced risk of antimicrobial resistance (AMR) by reducing the use of antibiotics. Statistical models suggest that these benefits of CT/NG POCTs are likely to result in longer-term cost savings to the wider health system.^22^ There were potential cost savings from reducing the number of patients referred from the spoke to the hub clinic (for SHS 1) which were not captured or assessed.

The introduction of any new technology within a service provides an opportunity to assess current practice and consider process changes to improve clinical care and rationalise resource use. Although there are national standards for STI testing,^6^ the way that clinics triage patients, the staffing grades and access to microscopy, differ between services. It is likely that clinics will use POC testing in different ways and in different patient groups according to where the clinic perceives the greatest need and/or benefit. In this study, no clinics chose to do an overhaul of the pathway, introduce POCT for other STIs or remove steps from the pathway as have been reported elsewhere.^11,16^ Once there is confidence in the new processes used for POC testing, some clinics may choose to adapt pathways further, for example, removing microscopy for most patients or using different staff grades for some tasks.

LTFU did not appear to be a problem at the participating SHSs and the cost of staff time and consumables used for follow-up (£1.54) made up only 3% of the overall pathway cost. However, in deciding where to use the POCT, all SHSs mentioned that they initially intended to use it for patients at high risk of LTFU, to prevent infections going untreated and to minimise efforts needed to contact patients requiring treatment. There is evidence from a small number of studies that CT/NG POCTs reduce LTFU rates.^27,28^ Our focus was on use of POCTs within specialist SHSs, where, in the United Kingdom, most STI testing takes place.^3^ Since POCTs provide an opportunity to test and treat at the same attendance using transportable equipment, they are ideally suited for high-risk populations in settings where LTFU is an issue or where testing would not normally be possible, including colleges, homeless shelters, festivals, and emergency services – and a number of studies have explored these.^10,15,23,29,30^

There are likely to be additional benefits of CT/NG POC testing which were not assessed, in future work it would be useful to model and explore the effect of receiving a positive (or negative) result sooner on onward transition, sexual behaviour, and anxiety levels in patients. POC testing may improve the partner notification (PN) process, something normally done by the patient, *via* text or social media, with support from clinical staff following a positive STI diagnosis.^6^ One SHS stated that PN was more effective during the patient’s attendance than over the phone. CT/NG POCTs may also lead to more rapid diagnosis of other STIs in symptomatic patients by rapidly ruling out chlamydia and gonorrhoea at the first attendance resulting in alternative infections being investigated and diagnosed sooner.^12,25^

This assessment provides further evidence that CT/NG POCTs used in SHSs benefit patients by reducing the time to results and treatment and reducing the unnecessary use of antibiotics.

## Supplemental Material

sj-docx-1-tai-10.1177_20499361211061645 – Supplemental material for Assessing the clinical impact and resource use of a 30-minute chlamydia and gonorrhoea point-of-care test at three sexual health services Click here for additional data file. Supplemental material, sj-docx-1-tai-10.1177_20499361211061645 for Assessing the clinical impact and resource use of a 30-minute chlamydia and gonorrhoea point-of-care test at three sexual health services by Susie Huntington, Georgie Weston and Elisabeth Adams in Therapeutic Advances in Infectious Disease
