# Supplementary material for: Assessing the clinical impact and resource use of a 30-minute chlamydia and gonorrhoea point-of-care test at three sexual health services
Source: Ther Adv Infect Dis. 2021 Dec 2;8:20499361211061645. doi: 10.1177/20499361211061645 (PMC8647227; doi:10.1177/20499361211061645)
Supplement: sj-docx-1-tai-10.1177_20499361211061645 – Supplemental material for Assessing the clinical impact and resource use of a 30-minute chlamydia and gonorrhoea point-of-care test at three sexual health services [file sj-docx-1-tai-10.1177_20499361211061645.docx]

**Supplementary Material**

**Assessing the clinical impact and resource use of a 30-minute chlamydia and gonorrhea point-of-care test at three sexual health services**

Contents

[Table S1. Overview of the approach used to map CT/NG testing and treatment pathways at sexual health services using semi-structured interviews 4](#_Toc37243246)

[Table S2. Overview of the approach used to collect resource use data in SHSs for standard and POC CT/NG testing and treatment pathways 5](#_Toc37243247)

[Table S3. Overview of the approach used for micro-costing resource use data in SHSs for standard and POC CT/NG testing and treatment pathways 7](#_Toc37243248)

[Table S4. Staff time unit costs – used to estimate average CT/NG testing and treatment pathway costs 9](#_Toc37243249)

[Table S5. Consumables unit costs – used to estimate average CT/NG testing and treatment pathway costs 10](#_Toc37243250)

[Table S6. Pathology and medication unit costs – used to estimate average CT/NG testing and treatment pathway costs 12](#_Toc37243251)

[Table S7. SHS 1: Summary of clinic specific patient groups and pathway adaptations 13](#_Toc37243252)

[Figure S1. SHS 1: Pathway adaptations – all patients attending the clinic for <25-year olds 14](#_Toc37243253)

[Table S8. SHS 1: Summary of data collection forms completed within clinic to assess patient clinical outcomes and resource use using standard CT/NG pathways compared to POC pathways 16](#_Toc37243254)

[Table S9. SHS 1: Patient numbers and risk groups for selected patient groups where clinical outcome and resource use data were collected to compare standard CT/NG pathways with POC pathways 17](#_Toc37243255)

[Table S10. SHS 1: CT and NG prevalence in patients with data collected to assess patient clinical outcomes and resource use using standard CT/NG pathways compared to POC pathways 18](#_Toc37243256)

[Table S11. SHS 1: Clinical outcomes for patients with data collected to compare the use of standard CT/NG pathways with POC pathways 19](#_Toc37243257)

[Table S12. SHS 1: Average pathway costs for standard and POC CT/NG testing and treatment pathways – calculated using resource use data collected in clinic for specific patient groups 21](#_Toc37243258)

[Table S13. SHS 2: Summary of clinic specific patient groups and pathway adaptations 23](#_Toc37243259)

[Figure S2. SHS 2: Pathway adaptations – Symptomatic patients 24](#_Toc37243260)

[Figure S3. SHS 2: Pathway adaptations – Asymptomatic - previous contact 26](#_Toc37243261)

[Figure S4. SHS 2: Pathway adaptations – women receiving emergency LARC 27](#_Toc37243262)

[Figure S5. SHS 2: Pathway adaptations – women receiving pre-planned LARC 29](#_Toc37243263)

[Table S14. SHS 2: Summary of data collection forms completed within clinic to assess patient clinical outcomes and resource use using standard CT/NG pathways compared to POC pathways 30](#_Toc37243264)

[Table S15. SHS 2: Patient numbers and risk groups for selected patient groups where clinical outcome and resource use data were collected to compare standard CT/NG pathways with POC pathways 31](#_Toc37243265)

[Table S16. SHS 2: CT and NG prevalence in patients with data collected to assess patient clinical outcomes and resource use using standard CT/NG pathways compared to POC pathways 32](#_Toc37243266)

[Table S17. SHS 2: Clinical outcomes for patients with data collected to compare the use of standard CT/NG pathways with POC pathways 33](#_Toc37243267)

[Table S18. SHS 2: Average pathway costs for standard and POC CT/NG testing and treatment pathways – calculated using resource use data collected in clinic for specific patient groups 35](#_Toc37243268)

[Table S19. SHS 3: Summary of clinic specific patient groups and pathway adaptations 36](#_Toc37243269)

[Figure S6. SHS 3: CT/NG treatment and testing pathways 37](#_Toc37243270)

[Table S20. SHS 3: Summary of data collection forms completed within clinic to assess patient clinical outcomes and resource use using standard CT/NG pathways compared to POC pathways 38](#_Toc37243271)

[Table S21. SHS 3: Patient numbers and risk groups for selected patient groups where clinical outcome and resource use data were collected to compare standard CT/NG pathways with POC pathways 39](#_Toc37243272)

[Table S22. SHS 3: CT and NG prevalence in patients with data collected to assess patient clinical outcomes and resource use using standard CT/NG pathways compared to POC pathways 40](#_Toc37243273)

[Table S23. SHS 3: Clinical outcomes for patients with data collected to compare the use of standard CT/NG pathways with POC pathways 41](#_Toc37243274)

[Table S24. SHS 3: Average pathway costs for standard and POC CT/NG testing and treatment pathways – calculated using resource use data collected in clinic for specific patient groups 43](#_Toc37243275)

## Table S1. Overview of the approach used to map CT/NG testing and treatment pathways at sexual health services using semi-structured interviews

| Approach | Adapting methods previously used to map pathways in SHSs ^1^, semi-structured interviews with service leads and/or clinical staff were held at each SHS attended by one/two members of the research team.  Interviews were audio recorded. |
| --- | --- |
| Patient groups | CT/NG testing and treatment pathways were mapped for each patient group where CT and NG laboratory testing was likely to be replaced with the CT/NG POCT. Potential pathways were identified by the clinics. |
| Pathway overview | Starting with existing pathways, a diagram of a typical pathway was used as the starting point. Large sticky notes were then used to map out key steps in the pathway including any branching (e.g. a branch for people with a positive test result and a branch for people with a negative test result). |
| Recording  resource use | Each step in the pathway was then revisited, recording on the sticky note the staff level and staff time for this step, plus the consumables used.  The number of patients in each pathway (per month) was recorded and where pathways branched, the number of patients flowing into each branch was recorded (typically an estimate or, in some cases, based on clinic data). |
| Adapting for POC | After discussing how the pathway could be adapted for the POCT, the mapping process was repeated for the (as yet) hypothetical POC pathway. |
| Summarising the pathway | Following the interview, summary diagrams were created in Excel for each pathway – listing the resource use for each step. These were emailed to the main contact at each clinic for confirmation and were later adapted (simplified) for this paper. |

CT, chlamydia; NG, gonorrhoea; POCT, point-of-care test; SHS, sexual health service.

## Table S2. Overview of the approach used to collect resource use data in SHSs for standard and POC CT/NG testing and treatment pathways

| Approach | Following pathway mapping using semi-structured interviews (**Table S1**), data collection (paper) forms were developed for use by clinic staff to record data on staff time, staff level and consumables. |
| --- | --- |
| Developing the data collection forms | Separate forms were developed for data collection: at first/return attendance (Form A and Form B, respectively); additional tasks performed during an attendance (Form C); additional tasks performed following an attendance (Form D); outcomes at 30-days after first attendance (Form E) and information on clinic staff paygrade (Form F). The data collected on each form are listed in **Table 1** in the main body of the text.  Forms were designed to be quick to complete and were a maximum of one double-sided A4 sheet. Staff initials and paygrade were collected. No patient identifying data were collected.  The wording and content of forms was refined following review by project advisors and the main contact at each participating clinic. |
| Data collection process | Forms for the same patient were linked using a unique 4-digit project ID. Four stickers for each project ID were taken in person or posted to clinics – additional copies were sent if requested or, in some cases, staff wrote the project ID on any additional forms.  A project ID sticker was stuck on each form relating to a patient.  There was space at the top of forms A-D to record the patient’s Clinic ID – so that clinics could easily link the form to the patient – this section of the form was removed by the clinic prior to forms being sent for data analysis. |
| Training and support | An information pack explaining the data collection process was provided. Any staff member asked to record data received training in person either by the lead contact at the clinic or by a visiting member of the research team. Additional support was offered and given via email and/or phone. |

Table continued on next page.

**Table S2.** Continued from previous page

CT, chlamydia; NG, gonorrhoea; POCT, point-of-care test; SHS, sexual health service.

| Resources provided | All data collection forms, project ID stickers and information packs were either taken in person or posted to clinics. Any additional forms requested were posted or sent as a pdf to be printed by the clinic. |
| --- | --- |
| Data collection duration | The clinic decided in advance, based on practical considerations, either the duration or the number of patients for whom they would complete forms.  The 30-day follow up form (Form E) was completed for all patients with a Form A by a member of the SHS team extracting data from each patient’s electronic record ≥30-days after the last Form A was complete.  The data collection was repeated for a similar duration or number of patients once the POC testing was in use at the clinic. |
| Returning completed forms | Once all the forms were complete, parts of the form containing the clinic ID was removed. Forms were then returned to the research team either by collection in person or courier. |

## Table S3. Overview of the approach used for micro-costing resource use data in SHSs for standard and POC CT/NG testing and treatment pathways

Continued on next page.

| Approach | The average cost of each pathway per patient for each patient group was calculated using a micro-costing approach. This combines the cost of staff time, with the cost of consumables (including consumables used for sample collection), CT/NG diagnostics and medication, for each attendance plus any follow-up and return appointment. Staff cost was calculated using a combined average of the average number of minutes for each staff grade multiplied by the cost per minute (See **Table S4**).  If, for example, data were collected for 20 patients and only one patient had a return appointment for treatment which cost £20 (staff time plus consumables), this cost would be split across all 20 patients – creating an average cost of £1 for the return appointment. Combined with the average cost of the first attendance and follow-up, this produced a total average pathway cost per patient. |
| --- | --- |
| Assumptions | If someone was reported to have returned for treatment (on their Form E) but there was no Form B completed for the return visit, the average cost of a return visit for treatment of the same infection was used (otherwise the average cost of this pathway would be underestimated).  If there was no form C or form D for a patient, it was assumed that no additional resources were used during or after the visit. |

**Table S3.** Continued from previous page

| Inclusions and exclusions | The cost of return appointments was included in the micro-costing calculations even when the return visit was not specifically for CT/NG treatment. The rationale being, that POC testing might reduce return appointments for other reasons as well, for example, a patient with symptoms testing negative for CT/NG at their first attendance would have further tests at that appointment instead of returning at a later time, when symptoms persist – as would be the case in standard care.  The cost of quality control (QC) was not considered. The Binx io™ platform does not require daily QC testing – and the level of QC testing is likely to differ according to local clinic POC requirements, whether the platform is moved from one location to another and the test throughput.  The staff time used for taking a blood sample for HIV and syphilis testing was included in the pathway as the time spent discussing these tests and taking the sample would form part of the initial consultation and could be affected with changes to the pathway – although the decision whether or not to test for HIV and syphilis and the resources used would not be affected by the CT/NG testing.  The cost of diagnostic tests for these infections and the cost of treating these infections would be unaffected by introduction of the CT/NG POC test and was not included in the pathway cost.  Test of cure (TOC) for NG was not included in pathway costs, since it was assumed that this would not be affected by the use of a POC testing at the initial clinic visit and because clinics stated that they would be unlikely to use POC testing for NG TOC. |
| --- | --- |

CT, chlamydia; QC quality control; NG, gonorrhoea; POC, point of care; TOC, test of cure.

## Table S4. Staff time unit costs – used to estimate average CT/NG testing and treatment pathway costs

| **Staff type** |  | **Cost per minute (£)** | **Source** | **Note** |
| --- | --- | --- | --- | --- |
|  |  |  |  |  |
| Admin/Clerical - Band 3 |  | 0.45 | Estimate | Based on the estimated cost of £27/hr (Band 3 cost not published in PSSRU 2018 ^2^) |
| Admin/Clerical - Band 4 |  | 0.52 | PSSRU 2018 ^2^ | Based on £31/hr for hospital-based scientific and professional staff |
| Admin/Clerical - Band 5 |  | 0.57 | PSSRU 2018 ^2^ | Based on £34/hr for hospital-based scientific and professional staff |
| Health Advisor - Band 5 |  | 0.57 | PSSRU 2018 ^2^ | Based on £34/hr for hospital-based scientific and professional staff |
| Health Advisor - Band 6 |  | 0.75 | PSSRU 2018 ^2^ | Based on £45/hr for hospital-based scientific and professional staff |
| Health Advisor - Band 7 |  | 0.92 | PSSRU 2018 ^2^ | Based on £55/hr for hospital-based scientific and professional staff |
| Healthcare Assistant - Band 3 |  | 0.45 | Estimate | Based on the estimated cost of £27/hr (equivalent to band 3 admin cost) |
| Healthcare Assistant - Band 4 |  | 0.47 | Estimate | Based on the estimated cost of £28/hr (equivalent to band 4 hospital-based nurse) |
| Nurse Band 5 |  | 0.62 | PSSRU 2018 ^2^ | Based on £37/hr for hospital-based nurses |
| Nurse Band 6 |  | 0.75 | PSSRU 2018 ^2^ | Based on £45/hr for hospital-based nurses |
| Nurse Band 7 |  | 0.90 | PSSRU 2018 ^2^ | Based on £54/hr for hospital-based nurses |
| Nurse Band 8a |  | 1.07 | PSSRU 2018 ^2^ | Based on £64/hr for hospital-based nurses |
| Registrar doctor |  | 0.72 | PSSRU 2018 ^2^ | Based on £43/hr for hospital-based doctors including qualification costs |
| Associate specialist doctor |  | 1.75 | PSSRU 2018 ^2^ | Based on £105/hr for hospital-based doctors including qualification costs |
| Consultant medical doctor |  | 1.80 | PSSRU 2018 ^2^ | Based on £108/hr for hospital-based doctors including qualification costs |
|  |  |  |  |  |

CT, chlamydia; Hr, hour; NG, gonorrhoea; PSSRU, Personal Social Services Research Unit. Costs published in PSSRU ^2^ take into account average working time, average salary, national insurance and superannuation, annual training and qualification costs, overheads, and the ratio of direct to patient-related time (for clinical staff). These are national average costs which take into account the higher costs in London and lower costs outside London.

## Table S5. Consumables unit costs – used to estimate average CT/NG testing and treatment pathway costs

| **Item** | **Cost per unit (£)** | **Unit** | **Source** | **Note** |
| --- | --- | --- | --- | --- |
| Paper form/letter/leaflet | 0.10 | Item | ^3^ | Assumed no inflation from 2016 cost |
| Phone call | 0.07 | Minute | ^3^ | Assumed no inflation from 2016 cost |
| Text message | 0.10 | Item | ^3^ | Assumed no inflation from 2016 cost |
| Email | 0.00 | Item | ^3^ | Assumed that there is no cost |
| Partner notification slip | 0.06 | Item | ^3^ | Assumed no inflation from 2016 cost |
| Blood tube | 0.13 | Item | ^3^ | Inflated by 3.5% from published 2016 unit costs |
| Cotton Wool | 0.02 | Item | ^3^ | Inflated by 3.5% from published 2016 unit costs |
| Disposable gloves (pair) | 0.06 | Item | ^3^ | Inflated by 3.5% from published 2016 unit costs |
| Laboratory request form with bag | 0.10 | Item | ^3^ | Inflated by 3.5% from published 2016 unit costs |
| Needle (sample collection) | 0.14 | Item | ^3^ | Inflated by 3.5% from published 2016 unit costs |
| Plaster | 0.05 | Item | ^3^ | Inflated by 3.5% from published 2016 unit costs |
| Couch roll | 0.08 | Item | ^3^ | Inflated by 3.5% from published 2016 unit costs |
| Antiseptic wipe | 0.20 | Item | ^3^ | Inflated by 3.5% from published 2016 unit costs |
| Saline (solution for injection) | 0.21 | Item | BNF – 2018 ^4^ | Lowest NHS indicative price - based on £2.07 for ten 2ml ampoules. |
| Syringe | 0.09 | Item | ^3^ | Inflated by 3.5% from published 2016 unit costs |
| Transport tube | 0.13 | Item | ^3^ | Inflated by 3.5% from published 2016 unit costs |
| Vacutainer | 0.17 | Item | ^3^ | Inflated by 3.5% from published 2016 unit costs |
| Sample collection instructions | 0.06 | Item | ^3^ | Inflated by 3.5% from published 2016 unit costs |
| Transport tube | 0.13 | Item | ^3^ | Inflated by 3.5% from published 2016 unit costs |
| Sample collection urine pot/container | 0.21 | Item | ^3^ | Inflated by 3.5% from published 2016 unit costs |
| Handwashing (soap and paper towels) | 0.08 | Item | ^3^ | Estimate |
| Vulvo-vaginal swab | 0.17 | Item | ^3^ | Inflated by 3.5% from published 2016 unit costs |
| Culture swab (NG) | 1.08 | Item | ^3^ | Inflated by 3.5% from published 2016 unit costs |

Table continued on next page.

**Table S5.** Continued from previous page.

| **Item** | **Cost per unit (£)** | **Unit** | **Source** | **Note** |
| --- | --- | --- | --- | --- |
| Swab | 0.11 | Item | ^3^ | Inflated by 3.5% from published 2016 unit costs |
| Sample collection plate | 1.08 | Item | ^3^ | Inflated by 3.5% from published 2016 unit costs |
| Needle (to administer treatment) | 0.14 | Item | ^3^ | Assumed same cost as needle for blood sample collection |
| Disposable tourniquet | 0.11 | Item |  | Lowest cost found in online search |
| Speculum | 0.87 | Item | ^3^ | Inflated by 3.5% from published 2016 unit costs |
| Lubricant | 0.31 | Application | ^3^ | Inflated by 3.5% from published 2016 unit costs |
| Microscopy test (bundled cost)^1^ | 0.48 | Item | ^3^ | Inflated by 3.5% from published 2016 unit costs |
| Female condom | 0.10 | Item | ^3^ | Inflated by 3.5% from published 2016 unit costs |
| Male condom | 0.10 | Item | ^3^ | Inflated by 3.5% from published 2016 unit costs |

CT, chlamydia; NG, gonorrhoea; NHS, National Health Service. The 2016 costs are the most recent costs published and used in Sexual Health Tariffs ^3^ (last accessed online 27^th^ March 2019).

^1^ The individual consumables used for microscopy were not itemised – instead a combined cost was used which included blotting paper, gram stain, immersion oil, loops and microscope slide.

## Table S6. Pathology and medication unit costs – used to estimate average CT/NG testing and treatment pathway costs

| **Item** | **Cost per unit (£)** | **Unit** | **Source** | **Note** |
| --- | --- | --- | --- | --- |
| CT/NG POC test | 23.00 | 1 | Estimate | This incorporates a £2 discount as the kit includes sample collection kit but sample collection kit was still included in micro-costing.^1^ |
| CT/NG lab test | 12.51 | 1 | ^3^ | Assumed no inflation from 2016 cost |
| Azithromycin (1g oral) | 0.33 | course | BNF - 2018 ^4^ | Lowest NHS indicative price |
| Azithromycin (extended dose) | 1.00 | course | BNF - 2018 ^4^ | Lowest NHS indicative price |
| Doxycycline (twice daily for 7 days) | 8.59 | course | BNF - 2018 ^4^ | Lowest NHS indicative price |
| Ceftriaxone (1g IM) | 9.58 | course | BNF - 2018 ^4^ | Lowest NHS indicative price |
| Ceftriaxone (1500mg IM) | 4.80 | course | BNF - 2018 ^4^ | Lowest NHS indicative price |

BNF, British National Formulary; CT, chlamydia; HIV, human immunodeficiency virus; NG, gonorrhoea; NHS, National Health Service. POC, point of care.

^1^It was assumed that the cost of rental and maintenance of the platform and quality assurance are incorporated into the per cartridge cost - as is common practice with many POC devices.

Costs are taken from London Sexual Health Tariff 2016 published costs ^3^. Although data on HIV, syphilis and NG culture were collected, the cost of these tests was not included in the micro-costing calculations.

Most CT/NG lab assays are not licensed for testing rectal swabs. As is currently the case with lab testing for CT/NG, it is likely that clinics will choose to test rectal samples off licence following internal validation.

## Table S7. SHS 1 Summary of clinic specific patient groups and pathway adaptations

| **Setting**  This SHS is located in London in an ethnically diverse residential neighbourhood. The borough has high levels of deprivation, being within the UK’s lowest deprivation quintile and around a quarter of the population are <19 years old. |
| --- |
| **SHS provision**  Sexual health and contraception are provided at four clinics run by the same NHS trust. Data were collected at two locations. Many of the clinical staff work at both.   1. Clinic A is a walk-in clinic for <25-year olds located within a Health and Leisure Centre. It is an open access nurse led service for young people including <16s offered two-days/week 3pm-7pm. 2. Clinic B is a walk-in SHS open 3-days/week for people of all ages, including young people.   Both sites provide STI testing and contraception services. Neither has microscopy on site. Patients with suspected gonorrhoea (NG) or *Trichomonas vaginalis* (TV) infection are referred to a larger (hub) clinic, located 5-miles from Clinic A and 3-miles from Clinic B, which runs a service 6-days/week with walk-in and booked appointments for people of all ages.  Free home STI testing kits are available from all three services or can be ordered online. |
| **Adapting the CT/NG testing and treatment pathway**  The SHS was interested in using the CT/NG POCT at two of its spoke clinics (where microscopy is not available). Initially, they wanted to use the POCT at Clinic B for contacts only, but subsequently decided to offer it to more patient groups at this clinic.  Also, they did not initially intend to offer POC testing at the hub clinic but due to the success of using it at the smaller clinics, POC testing was offered to some patients at the hub clinic and POC pathway data were collected for 13 patients. However, these were excluded, since no data on standard pathways had been collected for comparison. |
| **Resource use data collection**  The SHS chose to complete forms for approximately 40 patients for the standard and POC pathways. Forms (for patients first attendance) were completed in June-July 2019 for the standard pathways and in October 2019 for the POC pathways. |

## Figure S1. SHS 1. Pathway adaptations – all patients attending the clinic for <25-year olds

**Standard pathway**

**POC pathway**

**Summary**

This pathway is for all patients attending a walk-in clinic for young people. At first attendance, following meet and greet and assessment, patients with suspected NG or TV are referred to the larger hub clinic where microscopy is available. Of those not referred, some patients are given treatment for CT based on risk and symptoms. All have samples taken (swab/urine) and sent for CT/NG laboratory testing. Patients receive their test results by text. Those who had received treatment at their first visit receive a follow-up call to check drug adherence and that symptoms have cleared. Patients with a positive result who did not receive treatment at their first visit return for treatment and partner notification and then subsequently receive a treatment follow-up call.

In the POC pathway, patients self-collect a sample prior to their discussion with the nurse/doctor. If the result is positive, they receive treatment. Patients have blood tests send for laboratory testing and receive the results by text. Those who received treatment at their first visit subsequently receive a treatment follow-up call.

The pathway at Clinic B is similar. At the meet and greet step, a nurse triages patients, assessing their reason for attendance. At this stage, any asymptomatic contacts are encouraged to use online testing services.

## Table S8. SHS 1: Summary of data collection forms completed within clinic to assess patient clinical outcomes and resource use using standard CT/NG pathways compared to POC pathways

| Data collection form | | Standard CT/NG pathway | | | POC CT/NG pathway | | |
| --- | --- | --- | --- | --- | --- | --- | --- |
|  |  | Forms completed | Included | Excluded | Forms completed | Included | Excluded |
| Form A | First attendance | 89 | 38 | 51 | 48 | 46 | 2 |
| Form B | Return attendance | 9 | 8 | 1 | 6 | 6 | 0 |
| Form C | Additional tasks during attendance | 89 | 38 | 51 | 51 | 48 | 3 |
| Form D | Additional tasks following attendance | 74 | 56 | 18 | 0 | 0 | 0 |
| Form E | 30-day follow up | 38 | 38 | 0 | 49 | 47 | 2 |

The data collected on each form are listed in **Table 1** in the main body of the paper.

Forms were excluded if no CT/NG testing was performed or where resource use data were missing. Additional forms collected at the larger hub clinic for the POC pathway only were excluded and are not included in this table. For Clinic A, 22 Form A and Form C and 7 Form D were excluded for the standard pathway and 1 form A, C and E were exclude for POC pathways. For Clinic B, 29 Form A and Form C, 1 Form B and 11 Form D were excluded for the standard pathway and 1 Form A, 2 Form C and 1 Form E were excluded.

## Table S9. SHS 1: Patient numbers and risk groups for selected patient groups where clinical outcome and resource use data were collected to compare standard CT/NG pathways with POC pathways

| Sexual risk group | Standard CT/NG pathway | | | | | | | | | | | | POC CT/NG pathway | | | | | | | | | | | | |
| --- | --- | --- | --- | --- | --- | --- | --- | --- | --- | --- | --- | --- | --- | --- | --- | --- | --- | --- | --- | --- | --- | --- | --- | --- | --- |
|  | Asymptomatic | | Contact Asymptomatic | | Contra-ception | | Symptomatic | | Contact Symptomatic | | Total | | Asymptomatic | | Contact Asymptomatic | | | Contraception | | Symptomatic | | Contact Symptomatic | | Total | |
| Total | 15 | | 1 | | 11 | | 8 | | 3 | | 38 | | 1 | | 10 | | | 2 | | 29 | | 4 | | 46 | |
| Women | 7 | (47%) | 0 | (0%) | 7 | (64%) | 2 | (25%) | 2 | (64%) | 18 | (47%) | 1 | (100%) | | 4 | (40%) | 2 | (100%) | 7 | (24%) | 2 | (50%) | 16 | (35%) |
| MSM | 0 | (0%) | 0 | (0%) | 0 | (0%) | 1 | (13%) | 0 | (0%) | 1 | (3%) | 0 | (0%) | | 1 | (10%) | 0 | (0%) | 3 | (10%) | 0 | (0%) | 4 | (9%) |
| Heterosexual men | 5 | (33%) | 0 | (0%) | 2 | (18%) | 1 | (13%) | 0 | (0%) | 8 | (21%) | 0 | (0%) | | 3 | (30%) | 0 | (0%) | 17 | (59%) | 2 | (50%) | 22 | (48%) |
| Other/not reported | 3 | (20%) | 1 | (100%) | 2 | (18%) | 4 | (50%) | 1 | (33%) | 11 | (29%) | 0 | (0%) | | 2 | (20%) | 0 | (0%) | 2 | (7%) | 0 | (0%) | 4 | (9%) |

CT, chlamydia; MSM, men who have sex with men; NG, gonorrhoea; POC point-of-care.

For Clinic A, first attendance standard pathway data were collected over a 2-week period and POC pathway data were collected over a 2.6-week period. For Clinic B, standard pathway data were collected over a 2-week period and POC pathway data were collected over a 1.4-week period.

## Table S10. SHS 1: CT and NG prevalence in patients with data collected to assess patient clinical outcomes and resource use using standard CT/NG pathways compared to POC pathways

| Diagnosed  infection | Standard CT/NG pathway | | | | | | | | | | | | POC CT/NG pathway | | | | | | | | | | | |
| --- | --- | --- | --- | --- | --- | --- | --- | --- | --- | --- | --- | --- | --- | --- | --- | --- | --- | --- | --- | --- | --- | --- | --- | --- |
|  | Asymptomatic | | Contact Asymptomatic | | Contraception | | Symptomatic | | Contact Symptomatic | | Total | | Asymptomatic | | Contact Asymptomatic | | Contraception | | Symptomatic | | Contact Symptomatic | | Total | |
| Total | 15 | | 1 | | 11 | | 8 | | 3 | | 38 | | 1 | | 10 | | 2 | | 29 | | 4 | | 46 | |
| CT infection^1^ | 4 | (27%) | 1 | (100%) | 3 | (27%) | 1 | (13%) | 2 | (67%) | 11 | (29%) | 0 | (0%) | 5 | (50%) | 0 | (0%) | 8 | (28%) | 0 | (0%) | 13 | (28%) |
| NG infection^1^ | 0 | (0%) | 0 | (0%) | 0 | (0%) | 1 | (13%) | 1 | (33%) | 2 | (5%) | 0 | (0%) | 0 | (0%) | 0 | (0%) | 2 | (7%) | 1 | (25%) | 3 | (7%) |
| CT+NG  co-infection | 0 | (0%) | 0 | (0%) | 0 | (0%) | 0 | (0%) | 1 | (33%) | 1 | (3%) | 0 | (0%) | 0 | (0%) | 0 | (0%) | 1 | (3%) | 0 | (0%) | 1 | (2%) |

CT, chlamydia; NG, gonorrhoea; POC point-of-care.

^1^Includes those with CT/NG co-infection.

This table includes only infections diagnosed using molecular testing (lab or POCT).

## Table S11. SHS 1: Clinical outcomes for patients with data collected to compare the use of standard CT/NG pathways with POC pathways

| Clinical outcome  (within 30-days) | Standard CT/NG pathway | | | | | | | | | | | | POC CT/NG pathway | | | | | | | | | | | | |
| --- | --- | --- | --- | --- | --- | --- | --- | --- | --- | --- | --- | --- | --- | --- | --- | --- | --- | --- | --- | --- | --- | --- | --- | --- | --- |
|  | Asymptomatic | | Contact asymptomatic | | Contraception | | Symptomatic | | Contact symptomatic | | Total | | Asymptomatic | | Contacts asymptomatic | | Contraception | | | Symptomatic | | Contact symptomatic | | Total | |
| Total | 15 | | 1 | | 11 | | 8 | | 3 | | 38 | | 1 | | 10 | | 2 | | | 29 | | 4 | | 46 | |
| CT positive | 4 | (27%) | 1 | (100%) | 3 | (27%) | 1 | (13%) | 2 | (67%) | 11 | (29%) | 0 | (0%) | 5 | (50%) | 0 | (0%) | | 8 | (28%) | 0 | (0%) | 13 | (28%) |
| correctly treated at **first** appt | 0 | (0%) | 0 | (0%) | 0 | (0%) | 1 | (100%) | 2 | (100%) | 3 | (27%) | - | | 5 | (100%) | - | | | 8 | (100%) | - | | 13 | (100%) |
| correctly treated at **return** appt | 4 | (100%) | 1 | (100%) | 3 | (100%) | 0 | (0%) | 0 | (0%) | 8 | (73%) | - | | 0 | (0%) | - | | | 0 | (0%) | - | | 0 | (0%) |
| average wait (days) for trt^1^ | 11.3 | | 16.0 | | 17.0 | | 0.0 | | 0.0 | | 11.2 | | - | | 0.0 | | - | | | 0.0 | | - | | 0.0 | |
| CT negative | 11 | (73%) | 0 | (0%) | 8 | (73%) | 7 | (88%) | 1 | (33%) | 27 | (71%) | 1 | (100%) | 5 | (50%) | 2 | (100%) | | 21 | (72%) | 4 | (100%) | 33 | (72%) |
| unnecessary CT trt^2^ | 0 | (0%) | - | | 1 | (13%) | 0 | (0%) | 1 | (100%) | 2 | (7%) | 0 | (0%) | 0 | (0%) | 0 | (0%) | | 1 | (5%) | 1 | (25%) | 2 | (6%) |
| NG positive | 0 | (0%) | 0 | (0%) | 0 | (0%) | 1 | (13%) | 1 | (33%) | 2 | (5%) | 0 | (0%) | 0 | (0%) | 0 | (0%) | | 2 | (7%) | 1 | (25%) | 3 | (7%) |
| correctly treated at **first** appt | - | | - | | - | | 0 | (0%) | 1 | (100%) | 1 | (50%) | - | | - | | - | | | 2 | (100%) | 1 | (100%) | 3 | (100%) |
| correctly treated at **return** appt | - | | - | | - | | 1 | (100%) | 0 | (0%) | 1 | (50%) | - | | - | | - | | | 0 | (0%) | 0 | (0%) | 0 | (0%) |
| average wait (days) for trt^1^ | - | | - | | - | | 1.0 | | 0.0 | | 0.5 | | - | | - | | - | | | 0.0 | | 0.0 | | 0.0 | |
| NG negative | 15 | (100%) | 1 | (100%) | 11 | (100%) | 7 | (88%) | 2 | (67%) | 36 | (95%) | 1 | (100%) | 10 | (100%) | 2 | (100%) | | 27 | (93%) | 3 | (75%) | 43 | (93%) |
| unnecessary NG trt^2^ | 0 | (0%) | 0 | (0%) | 0 | (0%) | 0 | (0%) | 0 | (0%) | 0 | (0%) | 0 | (0%) | 0 | (0%) | 0 | (0%) | | 0 | (0%) | 0 | (0%) | 0 | (0%) |
|  |  |  |  |  |  |  |  |  |  | | | |  |  |  |  |  |  | |  |  |  |  |  |  |
| Days to CT/NG test result (mean) | 7.3 | | 9.0 | | 7.8 | | 8.4 | | 9.3 | | 7.9 | | 0.0 | | 0.0 | | 0.0 | | | 0.1 | | 0.0 | | 0.0 | |
| Return appt (for any reason) (%) | 4 | (27%) | 1 | (100%) | 2 | (12%) | 1 | (13%) | 0 | (0%) | 8 | (21%) | 0 | (0%) | 0 | (0%) | 1 | (50%) | | 4 | (14%) | 1 | (25%) | 5 | (11%) |
| Lost to follow-up^3^ | 0 | (0%) | 0 | (0%) | 0 | (0%) | 0 | (0%) | 0 | (0%) | 0 | (0%) | 0 | (0%) | 0 | (0%) | 0 | | (0%) | 0 | (0%) | 0 | (0%) | 0 | (0%) |

Appt, appointment/attendance; CT, chlamydia; NG, gonorrhoea; NGU, non-gonococcal urethritis; trt, treatment. Cells are left blank where not applicable.

^1^Average wait (days) for treatment is average wait from first attendance for patients diagnosed with CT or NG. ^2^Unnecessary treatment is treatment given at the first appt for CT/NG with a subsequent negative test result. This does not include patients treated for non-gonococcal urethritis (NGU). ^3^Lost to follow up refers to patients diagnosed with CT/NG who are not reported as having received treatment for the infection within the 30-day follow-up period.

In contacts POC pathway, one additional patient received treatment for NGU. In symptomatic POC pathway, one additional patient received treatment at their first appointment for NGU.

The unnecessary CT treatments in POC pathways were treatment given at first attendance despite a negative POC test – it is not clear whether this was because the result was thought to be a false negative i.e. because exposure was so recent – or because they were treated for NGU.

## Table S12. SHS 1. Average pathway costs for standard and POC CT/NG testing and treatment pathways – calculated using resource use data collected in clinic for specific patient groups

|  | Standard CT/NG pathway | | | | | | POC CT/NG pathway | | | | | |
| --- | --- | --- | --- | --- | --- | --- | --- | --- | --- | --- | --- | --- |
|  | Asymptomatic | Contacts asymptomatic | Contraception | Symptomatic | Contacts symptomatic | Total | Asymptomatic | Contacts asymptomatic | Contraception | Symptomatic | Contacts symptomatic | Total^2^ |
| Total number of patients | 15 | 1 | 11 | 8 | 3 | 38 | 1 | 10 | 2 | 29 | 4 | 46 |
| Staff time |  |  |  |  |  |  |  |  |  |  |  |  |
| 1^st^ visit main staff | £15.84 | £18.75 | £28.84 | £28.87 | £32.40 | £23.73 | £30.00 | £29.70 | £27.45 | £29.62 | £47.20 | £31.08 |
| 1^st^ visit additional staff | £1.86 | £1.35 | £1.63 | £1.80 | £0.90 | £1.69 | £3.60 | £2.57 | £2.93 | £1.80 | £6.41 | £2.46 |
| 2^nd^ visit main staff | £7.37 | £12.60 | £12.12 | £0.75 | £0.00 | £6.91 | £0.00 | £0.00 | No Data | £3.42 | £4.67 | £2.57 |
| 2^nd^ visit additional staff | £0.00 | £0.00 | £0.00 | £0.00 | £0.00 | £0.00 | £0.00 | £0.00 | No Data | £0.00 | £0.00 | £0.00 |
| Follow-up^1^ | £1.11 | £8.70 | £13.73 | £13.13 | £3.95 | £7.72 | £0.00 | £0.00 | £0.00 | £0.00 | £0.00 | £0.00 |
| Total staff time cost | £26.17 | £41.40 | £56.32 | £44.55 | £37.25 | £40.04 | £33.60 | £32.27 | £30.38 | £34.84 | £58.28 | £36.10 |
| Consumables |  |  |  |  |  |  |  |  |  |  |  |  |
| 1^st^ visit | £1.62 | £1.00 | £1.40 | £2.02 | £1.41 | £1.61 | £1.28 | £1.43 | £1.80 | £2.04 | £2.77 | £1.94 |
| 2^nd^ visit | £0.03 | £0.10 | £0.08 | £0.18 | £0.00 | £0.07 | £0.00 | £0.00 | No Data | £0.12 | £0.12 | £0.08 |
| Follow-up^1^ | £0.11 | £0.10 | £0.11 | £0.11 | £0.09 | £0.11 | £0.00 | £0.00 | £0.00 | £0.00 | £0.00 | £0.00 |
| Medication |  |  |  |  |  |  |  |  |  |  |  |  |
| 1^st^ visit | £0.00 | £0.00 | £0.78 | £1.08 | £8.92 | £1.16 | £0.00 | £4.30 | £0.00 | £2.80 | £4.54 | £3.10 |
| 2^nd^ visit | £1.96 | £0.33 | £0.97 | £1.33 | £0.00 | £1.34 | £0.00 | £0.00 | No Data | £0.59 | £0.00 | £0.37 |
| Diagnostics |  |  |  |  |  |  |  |  |  |  |  |  |
| 1^st^ visit | £12.51 | £12.51 | £12.51 | £12.51 | £12.51 | £12.51 | £23.00 | £23.00 | £23.00 | £23.00 | £23.00 | £23.00 |
| Average pathway cost | £42.40 | £55.44 | £72.17 | £61.76 | £60.18 | £56.84 | £57.88 | £61.00 | £55.18 | £63.40 | £88.71 | £64.60 |

^1^Follow-up refers to the tasks performed following the attendance such as processing and communicating test results.

^2^No data on resource use at return appointments were collected for the POC pathway for some patient groups who had a second attendance (reported on their 30-day follow-up form). To avoid under-estimating the cost of the POC pathway, the average cost per person per return visit (in standard pathway) was used to calculate the average cost of these return visits for the POC pathway and included in the total average cost of the pathway.

## Table S13. SHS 2: Summary of clinic specific patient groups and pathway adaptations

| **Setting**  This SHS is located in a market town and a university town within commuting distance of London. |
| --- |
| **SHS provision**  Data were collected at two sites run by the same NHS trust with many of the clinical staff working at both sites.  1) Clinic A is located within a hospital.  2) Clinic B is located within a health centre.  Both sites provide sexual health and contraceptive services with booked appointments and walk-in clinics throughout the week and are used by people living and working nearby as well as people from surrounding rural areas.  Both services provide free chlamydia postal testing which can be ordered online via the Terrance Higgins Trust^[[1]](#footnote-2)^. |
| **Adapting the CT/NG testing and treatment pathway**  The SHS was interested in using the CT/NG POCT in place of lab testing to reduce the time to results for symptomatic patients, asymptomatic patients who were (non-current) contacts and in women having long acting reversable contraception (LARC) fitted either as an emergency or booked in advance. Adaptations to the pathways are presented in **Figure S2-S5**. |
| **Resource use data collection**  The clinic chose to complete forms for 24 patients (in total) for the standard pathway and 24 for the POC pathway. Forms (for patients first attendance) were completed in May-July 2019 for the standard pathway and August-October 2019 for the POC pathway. |

## Figure S2. SHS 2. Pathway adaptations – Symptomatic patients

**Standard pathway**

**POC pathway**

**Summary**

The patient group is people attending a walk-in clinic with symptoms indicating an STI. At first attendance, microscopy is performed, and treatment is given to the patients – with the decision being based on risk, symptoms and microscopy results. Samples are sent for laboratory testing. Patients receive their test results by SMS. Those who had received treatment will have a follow-up call to check drug adherence and that symptoms have cleared. Patients with a positive result who did not receive treatment call the clinic to book a return appointment. They return to receive treatment and help with partner notification.

In the POC pathway, this return visit is unnecessary since patients are diagnosed and treated at their first attendance. In the POC pathway, patients still have lab test for other infections (HIV and syphilis) and patients positive for NG have samples sent for NG culture – for surveillance purposes. NG test of cure (TOC) is not included in this pathway as CT/NG POC testing would not be used for TOC.

## Figure S3. SHS 2. Pathway adaptations – Asymptomatic - previous contact

**Standard pathway**

**POC pathway**

**Summary**

The patient group includes patients who have been informed by a non-current sexual contact of an STI risk i.e. they are beyond the 2-week window period from exposure. Pathway changes are as with symptomatic patients **(Figure S2)** but there is no microscopy or examination.

## Figure S4. SHS 2. Pathway adaptations – women receiving emergency LARC

**Standard pathway**

**POC pathway**

**Summary**

The patient group is women attending who request emergency contraception and opt to have an intrauterine device (IUD) fitted. They are asymptomatic for an STI. In the standard pathway, samples are collected at the time of the coil (IUD) being fitted and sent for testing at the lab. Anyone who has a positive result then returns for treatment. In the POC pathway, the patient takes a sample – prior to the IDU fit. This is tested, while they wait and the appropriate treatment is given prior to the coil being fitted if the POCT is positive. Samples are sent for laboratory testing for other common STIs and for NG culture, if NG positive in POCT.

## Figure S5. SHS 2. Pathway adaptations – women receiving pre-planned LARC

**Standard pathway**

**POC pathway**

In standard care, women have bloods taken and a swab taken just prior to the IUD fit. These are sent for lab testing. Results are given by SMS, and if positive for CT/NG, women return for treatment.

In POC, women are tested for CT/NG at their first attendance. Women who are negative would then have IUD fitted at that appointment, women who are positive for CT or NG would receive treatment at that appointment and returning for the IUD fit at a subsequent appointment.

## Table S14. SHS 2: Summary of data collection forms completed within clinic to assess patient clinical outcomes and resource use using standard CT/NG pathways compared to POC pathways

| Data collection form | | Standard CT/NG pathway | | | POC CT/NG pathway | | |
| --- | --- | --- | --- | --- | --- | --- | --- |
|  |  | Forms completed | Included | Excluded | Forms completed | Included | Excluded |
| Form A | First attendance | 21 | 19 | 2 | 24 | 24 | 0 |
| Form B | Return attendance | 2 | 2 | 0 | 1 | 1 | 0 |
| Form C | Additional tasks during attendance | 21 | 17 | 4 | 33 | 33 | 0 |
| Form D | Additional tasks following attendance | 7 | 7 | 0 | 33 | 33 | 0 |
| Form E | 30-day follow up | 21 | 19 | 2 | 24 | 24 | 0 |

The data collected on each form are listed in **Table 1** in the main body of the paper.

Forms for two women receiving emergency LARC (standard pathway) were excluded as no CT/NG testing was performed.

## Table S15. SHS 2: Patient numbers and risk groups for selected patient groups where clinical outcome and resource use data were collected to compare standard CT/NG pathways with POC pathways

| Sexual risk group | Standard CT/NG pathway | | | | | | | | POC CT/NG pathway | | | | | | | |
| --- | --- | --- | --- | --- | --- | --- | --- | --- | --- | --- | --- | --- | --- | --- | --- | --- |
|  | Contacts asymptomatic | | Emergency LARC^1^ | | Symptomatic | | Total | | Contacts asymptomatic | | Emergency LARC^1^ | | Symptomatic | | Total | |
| Total | 12 | | 4 | | 3 | | 19 | | 8 | | 2 | | 14 | | 24 | |
| Women | 6 | (50%) | 4 | (100%) | 2 | (67%) | 12 | (63%) | 2 | (25%) | 2 | (100%) | 6 | (43%) | 10 | (42%) |
| MSM | 1 | (8%) | 0 | (0%) | 0 | (0%) | 1 | (5%) | 0 | (0%) | 0 | (0%) | 3 | (21%) | 3 | (13%) |
| Heterosexual men | 4 | (33%) | 0 | (0%) | 1 | (33%) | 5 | (26%) | 4 | (50%) | 0 | (0%) | 5 | (36%) | 9 | (38%) |
| Other/not reported | 1 | (8%) | 0 | (0%) | 0 | (0%) | 1 | (5%) | 2 | (25%) | 0 | (0%) | 0 | (0%) | 2 | (8%) |

CT, chlamydia; LARC, long-acting reversible contraception; MSM, men who have sex with men; NG, gonorrhoea; POC point-of-care.

^1^These are all unplanned (emergency) LARC patients. The planned LARC pathway was mapped, but no resource data were collected for this group.

Data were collected over 9-weeks for the standard pathway and 6.6 weeks for the POC pathway.

## Table S16. SHS 2: CT and NG prevalence in patients with data collected to assess patient clinical outcomes and resource use using standard CT/NG pathways compared to POC pathways

| Diagnosed infection | Standard CT/NG pathway | | | | | | | | POC CT/NG pathway | | | | | | | |
| --- | --- | --- | --- | --- | --- | --- | --- | --- | --- | --- | --- | --- | --- | --- | --- | --- |
|  | Contacts asymptomatic | | Emergency  LARC | | Symptomatic | | Total | | Contacts asymptomatic | | Emergency  LARC | | Symptomatic | | Total | |
| Total | 12 | | 4 | | 3 | | 19 | | 8 | | 2 | | 14 | | 24 | |
| CT infection | 2 | (17%) | 0 | (0%) | 1 | (33%) | 3 | (16%) | 2 | (25%) | 0 | (0%) | 5 | (36%) | 7 | (29%) |
| NG infection | 2 | (17%) | 0 | (0%) | 0 | (0%) | 2 | (11%) | 0 | (0%) | 0 | (0%) | 2 | (14%) | 2 | (8%) |
| CT+NG co-infection | 0 | (0%) | 0 | (0%) | 0 | (0%) | 0 | (0%) | 0 | (0%) | 0 | (0%) | 0 | (0%) | 0 | (0%) |

CT, chlamydia; LARC, long-acting reversible contraception; NG, gonorrhoea; POC point-of-care.

This table includes only infections diagnosed using molecular testing (lab or POCT).

## Table S17. SHS 2: Clinical outcomes for patients with data collected to compare the use of standard CT/NG pathways with POC pathways

| Clinical outcome (within 30-days) | Standard CT/NG pathway | | | | | | | | POC CT/NG pathway | | | | | | | |
| --- | --- | --- | --- | --- | --- | --- | --- | --- | --- | --- | --- | --- | --- | --- | --- | --- |
|  | Contacts asymptomatic | | Emergency LARC | | Symptomatic | | Total | | Contacts asymptomatic | | Emergency LARC | | Symptomatic | | Total | |
| Total | 12 | | 4 | | 3 | | 19 | | 8 | | 2 | | 14 | | 24 | |
| CT positive | 2 | (17%) | 0 | (0%) | 1 | (33%) | 3 | (16%) | 2 | (25%) | 0 | (0%) | 5 | (36%) | 7 | (29%) |
| correctly treated at **first** appt | 2 | (100%) | - | | 1 | (100%) | 3 | (100%) | 2 | (100%) | - | | 5 | (100%) | 7 | (100%) |
| correctly treated at **return** appt | 0 | (0%) | - | | 0 | (0%) | 0 | (0%) | 0 | (0%) | - | | 0 | (0%) | 0 | (0%) |
| average wait (days) for trt^1^ | 0 | | - | | 0 | | 0 | | 0 | | - | | 0 | | 0 | |
| CT negative | 10 | (83%) | 4 | (100%) | 2 | (67%) | 16 | (84%) | 6 | (75%) | 2 | (100%) | 9 | (64%) | 17 | (71%) |
| unnecessary CT trt^2^ | 4 | (40%) | 2 | (50%) | 0 | (0%) | 6 | (38%) | 1 | (17%) | 0 | (0%) | 1 | (11%) | 2 | (12%) |
| NG positive | 2 | (17%) | 0 | (0%) | 0 | (0%) | 2 | (11%) | 0 | (0%) | 0 | (0%) | 2 | (14%) | 2 | (8%) |
| correctly treated at **first** appt | 2 | (100%) | - | | - | | 2 | (100%) | - | | - | | 2 | (100%) | 2 | (100%) |
| correctly treated at **return** appt | 0 | (0%) | - | | - | | 0 | (0%) | - | | - | | 0 | (0%) | 0 | (0%) |
| average wait (days) for trt^1^ | 0 | | - | | - | | 0 | | - | | - | | 0 | | 0 | |
| NG negative | 10 | (83%) | 4 | (100%) | 3 | (100%) | 17 | (89%) | 8 | (100%) | 2 | (100%) | 12 | (86%) | 22 | (92%) |
| unnecessary NG trt^2^ | 0 | (0%) | 0 | (0%) | 0 | (0%) | 0 | (0%) | 0 | (0%) | 0 | (0%) | 0 | (0%) | 0 | (0%) |
|  |  |  |  |  |  |  |  |  |  |  |  |  |  |  |  |  |
| Days to CT/NG test result (mean) | 5.1 | | 4.7 | | 2.6 | | 4.2 | | 0.0 | | 0.0 | | 0.0 | | 0.0 | |
| Return appt (for any reason) (%) | 2 | (17%) | 0 | (0%) | 0 | (0%) | 2 | (11%) | 2 | (25%) | 0 | (0%) | 2 | (14%) | 4 | (17%) |
| Lost to follow-up^3^ | 0 | (0%) | - | | 0 | (0%) | 0 | (0%) | 0 | (0%) | - | | 0 | (0%) | 0 | (0%) |

Appt, appointment/attendance; CT, chlamydia; NG, gonorrhoea; NGU, non-gonococcal urethritis; trt, treatment. Cells are left blank where not applicable.

^1^Average wait (days) for treatment is average wait from first attendance for patients diagnosed with CT or NG. ^2^Unnecessary treatment is treatment given at the first appt for CT/NG with a subsequent negative test result. This does not include patients treated for non-gonococcal urethritis (NGU). ^3^Lost to follow up refers to patients diagnosed with CT/NG who are not reported as having received treatment for the infection within the 30-day follow-up period.

In contacts POC pathway, one additional patient received treatment for NGU. In symptomatic POC pathway, one additional patient received treatment at their first appointment for NGU.

The unnecessary CT treatments in POC pathways were treatment given at first attendance despite a negative POC test – it is not clear whether this was because the result was thought to be a false negative i.e. because exposure was so recent – or because they were treated for NGU.

| Costs | | Standard CT/NG pathway | | | | POC CT/NG pathway | | | |
| --- | --- | --- | --- | --- | --- | --- | --- | --- | --- |
|  |  | Contacts asymptomatic | Emergency LARC | Symptomatic | Total | Contacts asymptomatic | Emergency LARC | Symptomatic | Total^2^ |
| Total number of patients | | 12 | 4 | 3 | 19 | 8 | 2 | 14 | 24 |
| Staff time | 1^st^ visit main staff | £29.09 | £42.65 | £59.39 | £36.73 | £26.38 | £41.25 | £43.79 | £37.78 |
|  | 1^st^ visit additional staff | £1.87 | £16.52 | £6.01 | £5.61 | £13.65 | £3.38 | £9.57 | £9.10 |
|  | 2^nd^ visit main staff | £1.81 | £0.00 | £0.00 | £1.14 | No data | £0.00 | £0.31 | £1.63 |
|  | 2^nd^ visit additional staff | £0.00 | £0.00 | £0.00 | £0.00 | No data | £0.00 | £0.00 | £0.00 |
|  | Follow-up^1^ | £3.25 | £0.00 | £3.96 | £2.68 | £3.03 | £3.45 | £1.86 | £2.38 |
|  | Total staff time cost | £36.02 | £59.17 | £69.36 | £46.16 | £43.06 | £48.08 | £55.53 | £50.89 |
| Consumables | 1^st^ visit | £2.61 | £3.17 | £2.00 | £2.63 | £2.37 | £0.13 | £3.86 | £3.05 |
|  | 2^nd^ visit | £0.17 | £0.00 | £0.00 | £0.11 | No data | £0.00 | £0.00 | £0.13 |
|  | Follow-up^1^ | £0.34 | £0.00 | £0.41 | £0.28 | £0.19 | £0.07 | £0.35 | £0.27 |
| Medication | 1^st^ visit | £5.89 | £4.30 | £2.86 | £5.08 | £0.17 | £0.00 | £1.51 | £0.94 |
|  | 2^nd^ visit | £0.00 | £0.00 | £0.00 | £0.00 | No data | £0.00 | £0.00 | £0.00 |
| Diagnostics | 1^st^ visit | £12.63 | £12.63 | £12.83 | £12.66 | £23.06 | £23.00 | £23.38 | £23.24 |
| Total average pathway cost | | £57.66 | £79.27 | £87.46 | £66.91 | £64.91 | £71.28 | £84.63 | £78.52 |

## Table S18. SHS 2: Average pathway costs for standard and POC CT/NG testing and treatment pathways – calculated using resource use data collected in clinic for specific patient groups

^1^Follow-up refers to the tasks performed following the attendance such as processing and communicating test results.

^2^No data on resource use at return appointments were collected for the POC pathway for some patient groups who had a second attendance (reported on their 30-day follow-up form). To avoid under-estimating the cost of the POC pathway, the average cost per person per return visit (in standard pathway) was used to calculate the average cost of these return visits for the POC pathway and included in the total average cost of the pathway.

## Table S19. SHS 3: Summary of clinic specific patient groups and pathway adaptations

| **Setting**  This SHS is located near the south coast of England. |
| --- |
| **SHS provision**  The SHS is located within the main hospital serving the area. Diagnostic samples are sent off for lab testing with patients typically wait 2-weeks for a CT/NG test result. The staff mix is approximately: nurse band 6 (50%); nurse band 7 (21%); nurse band 8 (21%); doctor (associate level) (8%).  Most appointments must be booked in advance by phone – plus a weekly drop-in clinic for young people. Appointments are typically 20-minutes long.  Asymptomatic patients requesting an STI screen are referred to home testing kits which they can ordered online and are free of charge to patients^[[2]](#footnote-3)^. |
| **Adapting the CT/NG testing and treatment pathway**  The SHS was interested in using the CT/NG POCT in place of lab testing to reduce the time to results. They did not intend to use it as a rapid test i.e. the patient would not receive the result during their attendance. Therefore, the steps involved in CT/NG testing and treatment remained unaltered.  They initially intended to use the CT/NG POCT in symptomatic contacts, since 95% of patients are treated at their first appointment based on risk, symptoms and microscopy results. They later decided (for this project) to use the POCT in all patient groups, grouped as: symptomatic, contacts (asymptomatic) and asymptomatic. |
| **Resource use data collection**  The clinic chose to complete forms for 50 patients for the standard pathway and 50 for the POC pathway. Forms (for first attendance) were completed in April 2019 for the standard pathway and August-September 2019 for the POC pathway. |

## Figure S6. SHS 3. CT/NG treatment and testing pathways

**Standard Pathway**

The first attendance is a scheduled appointment pre-arranged by telephone.

For symptomatic contacts, the majority are treated at the first visit based on risk, results of examination and microscopy. An estimated 5% choose not to be treated at their first visit and have a scheduled appointment to return for treatment which is cancelled if the result is negative.

All patients with positive culture for NG are invited for an NG test of cure (TOC) – not shown here as CT/NG POCT test would not be used for TOC.

**POC Pathway**

There are no changes to the pathway with use of the CT/NG POC test – as the test is not used to give patients a rapid result during their attendance. Samples are tested for CT/NG in clinic but after the patient has left clinic. Additional samples are still sent to the lab for HIV, syphilis and NG culture (for surveillance purposes).

## Table S20. SHS 3. Summary of data collection forms completed within clinic to assess patient clinical outcomes and resource use using standard CT/NG pathways compared to POC pathways

| Data collection form | | Standard CT/NG pathway | | | POC CT/NG pathway | | |
| --- | --- | --- | --- | --- | --- | --- | --- |
|  |  | Forms completed | Included | Excluded | Forms completed | Included | Excluded |
| Form A | First attendance | 50 | 50 | 0 | 50 | 48 | 2 |
| Form B | Return attendance | 5 | 5 | 0 | 0 | 0 | 0 |
| Form C | Additional tasks during attendance | 97 | 94 | 0 | 91 | 89 | 2 |
| Form D | Additional tasks following attendance | 62 | 62 | 0 | 58 | 55 | 3 |
| Form E | 30-day follow up | 50 | 50 | 0 | 50 | 48 | 2 |

The data collected on each form are listed in **Table 1** in the main body of the paper.

Forms for two patients were excluded due to incomplete data relating to resource use.

## Table S21. SHS 3. Patient numbers and risk groups for selected patient groups where clinical outcome and resource use data were collected to compare standard CT/NG pathways with POC pathways

| Sexual risk group | Standard CT/NG pathway | | | | | | | | POC CT/NG pathway | | | | | | | |
| --- | --- | --- | --- | --- | --- | --- | --- | --- | --- | --- | --- | --- | --- | --- | --- | --- |
|  | Asymptomatic | | Contacts asymptomatic | | Symptomatic | | Total | | Asymptomatic | | Contacts asymptomatic | | Symptomatic | | Total | |
| Total | 26 | | 3 | | 21 | | 50 | | 29 | | 4 | | 15 | | 48 | |
| Women | 15 | (58%) | 2 | (67%) | 10 | (48%) | 27 | (54%) | 17 | (59%) | 2 | (50%) | 9 | (60%) | 28 | (58%) |
| MSM | 0 | (0%) | 0 | (0%) | 0 | (0%) | 0 | (0%) | 3 | (10%) | 1 | (25%) | 2 | (13%) | 6 | (13%) |
| Heterosexual men | 11 | (42%) | 0 | (0%) | 11 | (52%) | 22 | (44%) | 3 | (10%) | 1 | (25%) | 2 | (13%) | 6 | (13%) |
| Other/not reported | 0 | (0%) | 1 | (33%) | 0 | (0%) | 1 | (2%) | 6 | (21%) | 0 | (0%) | 2 | (13%) | 8 | (17%) |

CT, chlamydia; MSM, men who have sex with men; NG, gonorrhoea; POC point-of-care.

Data were collected over 2.6 weeks for standard pathway and 8 working days for POC pathway.

## Table S22. SHS 3. CT and NG prevalence in patients with data collected to assess patient clinical outcomes and resource use using standard CT/NG pathways compared to POC pathways

| Diagnosed infection | Standard CT/NG pathway | | | | | | | | POC CT/NG pathway | | | | | | | |
| --- | --- | --- | --- | --- | --- | --- | --- | --- | --- | --- | --- | --- | --- | --- | --- | --- |
|  | Asymptomatic | | Contacts asymptomatic | | Symptomatic | | Total | | Asymptomatic | | Contacts asymptomatic | | Symptomatic | | Total | |
| Total | 26 | | 3 | | 21 | | 50 | | 29 | | 4 | | 15 | | 48 | |
| CT infection | 1 | (4%) | 0 | (0%) | 0 | (0%) | 1 | (2%) | 1 | (3%) | 2 | (50%) | 0 | (0%) | 3 | (6%) |
| NG infection | 0 | (0%) | 0 | (0%) | 0 | (0%) | 0 | (0%) | 0 | (0%) | 0 | (0%) | 0 | (0%) | 0 | (0%) |
| CT+NG co-infection | 0 | (0%) | 0 | (0%) | 0 | (0%) | 0 | (0%) | 0 | (0%) | 0 | (0%) | 0 | (0%) | 0 | (0%) |

CT, chlamydia; NG, gonorrhoea; POC point-of-care.

This table includes only infections diagnosed using molecular testing (lab or POCT).

## Table S23. SHS 3. Clinical outcomes for patients with data collected to compare the use of standard CT/NG pathways with POC pathways

| Clinical outcome (within 30-days) | Standard CT/NG pathway | | | | | | | | POC CT/NG pathway^4^ | | | | | | | |
| --- | --- | --- | --- | --- | --- | --- | --- | --- | --- | --- | --- | --- | --- | --- | --- | --- |
|  | Asymptomatic | | Contacts asymptomatic | | Symptomatic | | Total | | Asymptomatic | | Contacts asymptomatic | | Symptomatic | | Total | |
| Total | 26 | | 3 | | 21 | | 50 | | 29 | | 4 | | 15 | | 48 | |
| CT positive | 1 | (4%) | 0 | (0%) | 0 | (0%) | 1 | (2%) | 1 | (3%) | 2 | (50%) | 0 | (0%) | 3 | (6%) |
| correctly treated at **first** appt | 1 | (100%) | - | | - | | 1 | (100%) | 0 | (0%) | 2 | (100%) | - | | 2 | (67%) |
| correctly treated at **return** appt | 0 | (0%) | - | | - | | 0 | (0%) | 1 | (100%) | 0 | (0%) | - | | 1 | (33%) |
| average wait (days) for trt^1^ | 0 | | - | | - | | 0 | | 0^5^ | | 0 | | - | | 0^5^ | |
| CT negative | 25 | (96%) | 3 | (100%) | 21 | (100%) | 49 | (98%) | 28 | (97%) | 2 | (50%) | 15 | (100%) | 45 | (94%) |
| unnecessary CT trt^2^ | 0 | (0%) | 3 | (100%) | 1 | (5%) | 4 | (8%) | 0 | (0%) | 1 | (50%) |  |  | 1 | (2%) |
| NG positive | 0 | (0%) | 0 | (0%) | 0 | (0%) | 0 | (0%) | 0 | (0%) | 0 | (0%) | 0 | (0%) | 0 | (0%) |
| correctly treated at **first** appt | - | | - | | - | | - | | - | | - | | - | | - | |
| correctly treated at **return** appt | - | | - | | - | | - | | - | | - | | - | | - | |
| average wait (days) for trt^1^ | - | | - | | - | | - | | - | | - | | - | | - | |
| NG negative | 26 | (100%) | 3 | (100%) | 21 | (100%) | 50 | (100%) | 29 | (100%) | 4 | (100%) | 15 | (100%) | 48 | (100%) |
| unnecessary NG trt^2^ | 0 | (0%) | 0 | (0%) | 0 | (0%) | 0 | (0%) | 0 | (0%) | 0 | (0%) | 0 | (0%) | 0 | (0%) |
|  |  |  |  |  |  |  |  |  |  |  |  |  |  |  |  |  |
| Days to CT/NG test result (mean) | 14.3 | | 16.3 | | 15.6 | | 15.5 | | 6.5 | | 3.3 | | 7.3 | | 6.5 | |
| Return appt (for any reason) (%) | 2 | (8%) | 0 | (0%) | 3 | (14%) | 5 | (10%) | 5 | (17%) | 1 | (25%) | 3 | (20%) | 9 | (19%) |
| Lost to follow-up^3^ | 0 | (0%) | 0 | (0%) | 0 | (0%) | 0 | (0%) | 0 | (0%) | 0 | (0%) | 0 | (0%) | 0 | (0%) |

Appt, appointment/attendance; CT, chlamydia; NG, gonorrhoea; trt, treatment. Cells are left blank where not applicable.

^1^Average wait (days) for treatment is average wait from first attendance for patients diagnosed with CT or NG. ^2^Unnecessary treatment is treatment given at the first appt for CT/NG with a subsequent negative test result. This does not include patients treated for non-gonococcal urethritis (NGU). ^3^Lost to follow up refers to patients diagnosed with CT/NG who are not reported as having received treatment for the infection within the 30-day follow-up period.

^4^ Although this is labelled as POC, samples were not always tested during the patient’s appointment – and therefore it was not a genuine POC pathway. ^5^For one patient at SHS 3, data were missing on the date the patient returned for CT treatment after receiving their CT/NG test result on the same day they attended.

|  | | Standard CT/NG pathway | | | | POC CT/NG pathway | | | |
| --- | --- | --- | --- | --- | --- | --- | --- | --- | --- |
|  |  | Asymptomatic | Contacts asymptomatic | Symptomatic | Total | Asymptomatic | Contacts asymptomatic | Symptomatic | Total^2^ |
| Total number of patients | | 26 | 3 | 21 | 50 | 29 | 4 | 15 | 48 |
| Staff time | 1^st^ visit main staff | £13.37 | £18.90 | £17.18 | £15.30 | £13.68 | £11.21 | £16.05 | £14.22 |
|  | 1^st^ visit additional staff | £4.54 | £1.41 | £3.98 | £4.12 | £4.34 | £7.28 | £4.54 | £4.65 |
|  | 2^nd^ visit main staff | £1.07 | £0.00 | £2.06 | £1.42 | No data | No data | No data | £2.67 |
|  | 2^nd^ visit additional staff | £0.00 | £0.00 | £0.00 | £0.00 | No data | No data | No data | £0.00 |
|  | Follow-up^1^ | £1.90 | £6.52 | £3.49 | £2.84 | £1.89 | £6.89 | £1.50 | £2.18 |
|  | Total staff time cost | £20.88 | £26.83 | £26.71 | £23.68 | £19.91 | £25.39 | £22.09 | £23.72 |
| Consumables | 1^st^ visit | £2.64 | £2.38 | £2.22 | £2.45 | £2.20 | £2.71 | £2.70 | £2.40 |
|  | 2^nd^ visit | £0.05 | £0.00 | £0.19 | £0.10 | No data | No data | No data | £0.19 |
|  | Follow-up^1^ | £0.17 | £0.68 | £0.26 | £0.24 | £0.22 | £0.58 | £0.30 | £0.28 |
| Medication | 1^st^ visit | £0.33 | £8.59 | £0.82 | £1.03 | £0.00 | £2.64 | £0.00 | £0.22 |
|  | 2^nd^ visit | £0.00 | £0.00 | £0.41 | £0.17 | No data | No data | No data | £0.32 |
| Diagnostics^3^ | 1^st^ visit | £12.51 | £12.51 | £12.67 | £12.58 | £23.00 | £23.00 | £23.10 | £23.03 |
| Total average pathway cost | | £36.58 | £50.99 | £43.27 | £40.25 | £45.33 | £54.32 | £48.19 | £50.15 |

## Table S24. SHS 3. Average pathway costs for standard and POC CT/NG testing and treatment pathways – calculated using resource use data collected in clinic for specific patient groups

^1^Follow-up refers to the tasks performed following the attendance such as processing and communicating test results. No form Cs were completed for any second attendance. ^2^No data on resource use at return appointments were collected for the POC pathway for the 9 patients who had second attendance (reported on their 30-day follow-up form). To avoid underestimating the cost of the POC pathway, the average cost per person per return visit (in standard pathway) was used to calculate the average cost of these return visits for the POC pathway and added to the total average cost of the pathway. ^3^CT/NG diagnostics and microscopy only, this does not include the cost of HIV and syphilis testing or NG culture.

**References**

1. Adams EJ, Ehrlich A, Turner KME, et al. Mapping patient pathways and estimating resource use for point of care versus standard testing and treatment of chlamydia and gonorrhoea in genitourinary medicine clinics in the UK. *BMJ Open* 2014; 4: e005322.

2. Curtis, L. & Burns, A. *(2018) Unit Costs of Health and Social Care 2018, Personal Social Services Research Unit, University of Kent, Canterbury*, https://www.pssru.ac.uk/project-pages/unit-costs/unit-costs-2018/.

3. Pathway Analytics. *Integrated Sexual Health Tariff - London ISHT 2017/2018. https://www.pathwayanalytics.com/*.

4. BNF: British National Formulary - NICE. https://bnf.nice.org.uk/.

1. <https://www.tht.org.uk/our-services/phone-and-post/free-chlamydia-postal-test-kits> [↑](#footnote-ref-2)
2. <https://www.freetest.me/> [↑](#footnote-ref-3)
